# Structure-function analysis of USP1: insights into the role of Ser313 phosphorylation site and the effect of cancer-associated mutations on autocleavage

**DOI:** 10.1186/s12943-015-0311-7

**Published:** 2015-02-06

**Authors:** Anne Olazabal-Herrero, Iraia García-Santisteban, Jose Antonio Rodríguez

**Affiliations:** Department of Genetics, Physical Anthropology and Animal Physiology, University of the Basque Country (UPV/EHU), Barrio Sarriena s/n, Leioa, 48940 Spain

## Abstract

**Background:**

In complex with its cofactor UAF1, the USP1 deubiquitinase plays an important role in cellular processes related to cancer, including the response to DNA damage. The USP1/UAF1 complex is emerging as a novel target in cancer therapy, but several aspects of its function and regulation remain to be further clarified. These include the role of the serine 313 phosphorylation site, the relative contribution of different USP1 sequence motifs to UAF1 binding, and the potential effect of cancer-associated mutations on USP1 regulation by autocleavage.

**Methods:**

We have generated a large set of USP1 structural variants, including a catalytically inactive form (C90S), non-phosphorylatable (S313A) and phosphomimetic (S313D) mutants, deletion mutants lacking potential UAF1 binding sites, a mutant (GG/AA) unable to undergo autocleavage at the well-characterized G670/G671 diglycine motif, and four USP1 mutants identified in tumor samples that cluster around this cleavage site (G667A, L669P, K673T and A676T). Using cell-based assays, we have determined the ability of these mutants to bind UAF1, to reverse DNA damage-induced monoubiquitination of PCNA, and to undergo autocleavage.

**Results:**

A non-phosphorylatable S313A mutant of USP1 retained the ability to bind UAF1 and to reverse PCNA ubiquitination in cell-based assays. Regardless of the presence of a phosphomimetic S313D mutation, deletion of USP1 fragment 420–520 disrupted UAF1 binding, as determined using a nuclear relocation assay. The UAF1 binding site in a second UAF1-interacting DUB, USP46, was mapped to a region homologous to USP1(420–520). Regarding USP1 autocleavage, co-expression of the C90S and GG/AA mutants did not result in cleavage, while the cancer-associated mutation L669P was found to reduce cleavage efficiency.

**Conclusions:**

USP1 phosphorylation at S313 is not critical for PCNA deubiquitination, neither for binding to UAF1 in a cellular environment. In this context, USP1 amino acid motif 420–520 is necessary and sufficient for UAF1 binding. This motif, and a homologous amino acid segment that mediates USP46 binding to UAF1, map to the Fingers sub-domain of these DUBs. On the other hand, our results support the view that USP1 autocleavage may occur in *cis,* and can be altered by a cancer-associated mutation.

**Electronic supplementary material:**

The online version of this article (doi:10.1186/s12943-015-0311-7) contains supplementary material, which is available to authorized users.

## Background

Ubiquitin Specific Protease 1 (USP1) is a human deubiquitinase (DUB) that plays an important role in the regulation of the cellular response to DNA damage and is also involved in the control of cell differentiation (reviewed in [1]). USP1 is a 785 amino acid protein, whose three-dimensional structure has not yet been solved. Structural analysis of other USP family members, such as USP7, has shown that the catalytic domain of these enzymes adopts a fold that resembles an open right hand with three sub-domains termed Fingers, Palm and Thumb [2,3]. A detailed sequence alignment analysis has further revealed that the USP core catalytic domain can be divided into six conserved boxes (boxes 1–6), and that several of these enzymes, including USP1, contain additional non-conserved domains inserted between the boxes that may play a regulatory role [4]. USP1 bears one of the largest catalytic domains within the USP family, which includes two inserted domains between boxes 2 and 3, and between boxes 5 and 6 [4].

One of the best-characterized functions of USP1 in the DNA damage response is as a regulator of Proliferating Cell Nuclear Antigen (PCNA) ubiquitination [5]. Following DNA damage that stalls progression of the replication fork, PCNA is monoubiquitinated to promote the recruitment of translesion synthesis (TLS) DNA polymerases, which can bypass the lesion [6]. USP1 deubiquitinates PCNA, thus contributing to prevent unscheduled recruitment of error-prone TLS DNA polymerases [5].

USP1 carries out its function in the context of a heterodimeric complex with its cofactor USP1-Associated Factor 1 (UAF1). UAF1 has been shown to stabilize USP1 [7] and to allosterically increase its catalytic activity, which is very low in the absence of the cofactor [7,8]. UAF1 also contributes to target USP1 to its nuclear substrates [9]. In addition to USP1, UAF1 also binds to and regulates the activity of two other members of the USP family, USP12 and USP46 [10].

Overexpression of USP1 has been reported in osteosarcoma [11] and non-small cell lung cancer (NSCLC) [1,12], among other cancer types. In addition, USP1 mutations have been identified, albeit at a low frequency, in tumor samples [13]. The functional effect of these cancer-associated USP1 mutations remains to be investigated. Importantly, several inhibitors of the USP1/UAF1 complex have been recently shown to act synergistically with cisplatin in cancer-derived cell lines [1,14,15], suggesting that this complex may represent a valid therapeutic target in cancer. The development and implementation of USP1-targeted therapies will benefit from a more detailed knowledge of how the function of this DUB is regulated, and how this regulation can be affected by cancer-related mutations.

Several regulatory mechanisms converge to determine the levels, localization and activity of USP1 (reviewed in [1]). These mechanisms include phosphorylation and autocleavage.

Phosphorylation at serine 313 (S313), within the first inserted domain, was initially reported to regulate cell cycle-dependent degradation of USP1. Thus, cyclin-dependent kinase 1 (CDK1)-mediated phosphorylation of S313 during M phase was shown to prolong the stability of USP1 presumably by preventing its degradation by the anaphase-promoting complex/cyclosome [16]. More recently, S313 phosphorylation has been reported to be critical for UAF1 interaction and USP1 catalytic activity *in vitro* [17]. In this study, using pull-down assays with purified recombinant proteins, the UAF1-binding motif was mapped to USP1 amino acid region 235–408 [17]. In contrast, using cell-based assays, we have mapped the UAF1-binding region in USP1 to a 100 amino acid motif comprising residues 420–520, which does not include the S313 phosphorylation site [18]. It is, therefore, necessary to clarify these conflicting results, and to determine to what extent these two amino acid motifs, and the S313 phosphorylation site, contribute to USP1/UAF1 interaction.

Another important regulatory mechanism involves the autocleavage of USP1 at a diglycine motif (G670/G671) located within its second inserted domain [5]. The cleavage event generates a longer N-terminal fragment (residues 1–671) and a shorter C-terminal fragment (residues 672–785), which are subsequently degraded by the proteasome [5,19]. It remains to be elucidated if USP1 autocleavage occurs in *cis* (intramolecularly) or in *trans* (intermolecularly) and, more importantly, if USP1 autocleavage could be altered by cancer-associated USP1 mutations that cluster around the G670/G671 motif.

In the present work, we use site-directed mutagenesis and cell-based functional assays to carry out a detailed structure-function analysis of human USP1. Our results indicate that the S313 phosphorylation site is not critical for USP1 binding to UAF1 or for PCNA deubiquitination in a cellular environment. Furthermore, we show that two homologous amino acid segments in USP1 (420–520) and USP46 (165–259), which are predicted to correspond to the Fingers sub-domains, mediate binding of these DUBs to UAF1. Moreover, we provide some experimental evidence suggesting that USP1 autocleavage may occur in *cis*. Finally, we identify a cancer-associated mutation in a residue adjacent to the cleavage site (L669P), that hampers USP1 autocleavage.

## Results

### UAF1 binds and stabilizes a non-phosphorylatable S313A mutant version of USP1 in cell-based assays

**Figure 1:**
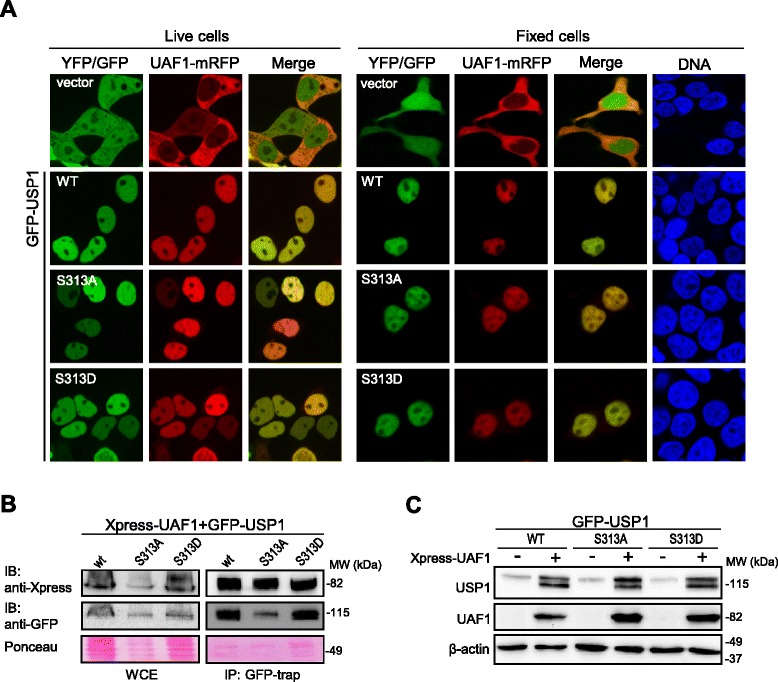
**UAF1 binds and stabilizes a non-phosphorylatable S313A mutant version of USP1 in cell-based assays. A**. Confocal images show representative examples of 293T cells co-expressing UAF1-mRFP with YFP (vector), GFP-USP1 wild type (WT), GFP-USP1^S313A^ or GFP-USP1^S313D^. Left panels show live cell images, whereas right panels show images of fixed cells. Fixed cells were counterstained with Hoechst to show the nuclei (DNA panels). UAF1-mRFP is cytoplasmic when co-expressed with YFP, but relocates to the nucleus when co-expressed with GFP-USP1 wild type, GFP-USP1^S313A^ and GFP-USP1^S313D^. **B**. Co-IP analysis of co-transfected 293T cells, showing that Xpress-UAF1 readily co-immunoprecipitates with GFP-USP1 wild type, GFP-USP1^S313A^ and GFP-USP1^S313D^. A section of the membrane stained with Ponceau is shown to gauge protein loading. WCE, whole cell extract. **C**. Immunoblot analysis of 293T cells transfected with GFP-USP1 wild type, GFP-USP1^S313A^ and GFP-USP1^S313D^, alone (−) or in combination with Xpress-UAF1 (+). Anti-GFP antibody was used to detect GFP-USP1 proteins and anti-Xpress antibody was used to detect Xpress-UAF1. β-actin was used as a loading control. UAF1 co-expression markedly increases the levels of GFP-USP1 wild type, GFP-USP1^S313A^ and GFP-USP1^S313D^ to a similar extent. The lower molecular weight band in (+) samples corresponds to the well-characterized N-terminal fragment that results from USP1 autocleavage at the G670/G671 diglycine motif (see below).

It has been recently reported that phosphorylation of USP1 at S313 is necessary for the interaction of this DUB with its cofactor UAF1 [17]. Since only *in vitro* evidence for S313 phosphorylation-regulated USP1/UAF1 interaction has been provided, we decided to evaluate the role that this phosphorylation site may have in regulating USP1 binding to UAF1 in a cellular context. To this end, we generated non-phosphorylatable (S313A) and phosphomimetic (S313D) mutant versions of USP1, and tested their ability to interact with UAF1 in cell-based assays. We have previously described a cellular relocation assay to evaluate USP1/UAF1 interaction [18]. This assay is based on the nuclear accumulation of UAF1 induced by USP1 co-expression and, by using fluorescently-tagged versions of these proteins, the results can be observed in live cells, before processing for microscopy analysis. As shown in Figure 1A, UAF1-mRFP was predominantly located in the cytoplasm of both live and fixed 293T cells when co-expressed with YFP (negative control). UAF1-mRFP was also cytoplasmic when expressed alone (Additional file 1A). Consistent with our previous report [18], UAF1-mRFP accumulated in the nucleus when co-expressed with GFP-USP1. Importantly, both GFP-USP1^S313A^ and GFP-USP1^S313D^ were able to relocate UAF1-mRFP to the nucleus, suggesting that both mutants are still able to interact with UAF1. Similar results were obtained when endogenous USP1 gene expression was down-regulated using siRNA (Additional file 1B,C). In line with the results of the relocation assay, co-immunoprecipitation (co-IP) analysis (Figure 1B) revealed that both GFP-USP1^S313A^ and GFP-USP1^S313D^ co-immunoprecipitated Xpress-UAF1 as efficiently as wild type GFP-USP1.

Next, we compared the effect of UAF1 expression on the stability of the different USP1 variants, since UAF1 has been shown to promote USP1 stabilization [7]. Indeed, a dramatic increase in the level GFP-USP1 was observed in 293T cells co-transfected with Xpress-UAF1 (Figure 1C). Of note, the increase in USP1 levels was accompanied by the appearance of a lower molecular weight band. As further discussed below, this band most likely corresponds to the amino-terminal fragment of USP1 resulting from its cleavage at the well-characterized G670/G671 autocleavage site [5]. No differences in UAF1-induced stabilization or cleavage were observed between the wild type USP1 and the S313A or S313D mutants.

The finding that the non-phosphorylatable S313A mutant version of USP1 binds and is stabilized by UAF1 in our cell-based assays indicates that phosphorylation at S313 is not critical for USP1/UAF1 complex formation in a cellular context.

### The non-phosphorylatable USP1^S313A^ mutant maintains its PCNA deubiquitinating activity

The S313A mutation has been reported to drastically reduce the catalytic activity of USP1 in *in vitro* assays using an artificial fluorogenic substrate [17]. We decided to test the effect of this mutation on the ability of USP1 to deubiquitinate a physiologically relevant substrate of this enzyme, monoubiquitinated PCNA (ubPCNA).

**Figure 2:**
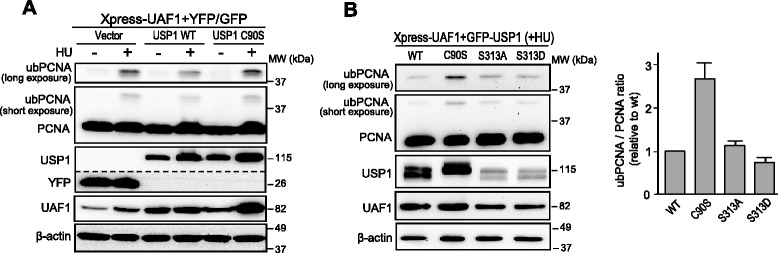
**USP1-mediated PCNA deubiquitination is not abrogated by the S313A mutation. A**. Immunoblot analysis of 293T cells co-transfected with Xpress-UAF1 and YFP-vector, GFP-USP1 wild type (WT) or the catalytically inactive GFP-USP1^C90S^. Cells were either left untreated (−), or were treated (+) with 4 mM hydroxyurea (HU) for 24 h. Using an anti-PCNA antibody, monoubiquitinated PCNA (ubPCNA) is detected as a band migrating slightly above the non-ubiquitinated form (PCNA). A short-exposure time image showing both PCNA and ubPCNA, as well as a cropped image showing only ubPCNA with longer exposure time are shown. The dotted line indicates that a panel is a composite of two images from a single exposure of the same gel. Expression of wild type GFP-USP1 decreased HU-induced PCNA monoubiquitination, whereas expression of GFP-USP1^C90S^ did not. Expression of β-actin was used as a control for equal loading of the protein samples. **B**. On the left, representative example of immunoblot analysis of 293T cells co-transfected with Xpress-UAF1 and GFP-USP1 wild type, GFP-USP1^C90S^, GFP-USP1^S313A^ or GFP-USP1^S313D^, and treated with 4 mM HU for 24 h. The ratio of ubiquitinated to non-ubiquitinated PCNA (ubPCNA/PCNA ratio) was determined by densitometry analysis of the immunoblot bands. The graph on the right shows the results of this analysis. The ubPCNA/PCNA ratio was similar in cells expressing wild type GFP-USP1, GFP-USP1^S313A^ and GFP-USP1^S313D^, but higher in cells expressing the catalytically inactive GFP-USP1^C90S^. The data represent the mean and SEM of 7 independent experiments.

As previously shown [5], we found that treatment of 293T cells with hydroxyurea (HU) induced PCNA monoubiquitination (Additional file 2), which was reduced by ectopic expression of wild type GFP-USP1, but not by expression of a catalytically inactive C90S mutant (Figure 2A). Next, 293T cells were co-transfected with plasmids encoding Xpress-UAF1 and GFP-USP1 wild type, GFP-USP1^C90S^, GFP-USP1^S313A^ or GFP-USP1^S313D^, and treated with HU. As shown in Figure 2B, both S313A and S313D mutants, but not the C90S mutant, were able to decrease HU-induced PCNA monoubiquitination. Nevertheless, the ratio of ubiquitinated to non-ubiquitinated PCNA (ubPCNA/PCNA ratio) was repeatedly observed in several independent experiments to be slightly higher in cells expressing GFP-USP1^S313A^ and slightly lower in cells expressing GFP-USP1^S313D^ in comparison to cells expressing wild type GFP-USP1. From these results, we conclude that phosphorylation at S313 is not necessary for USP1-mediated PCNA deubiquitination in HU-treated cells, although it might contribute to modulate this activity.

### Side-by-side comparison of two reported UAF1-binding sites in USP1 using the nuclear relocation assay

**Figure 3:**
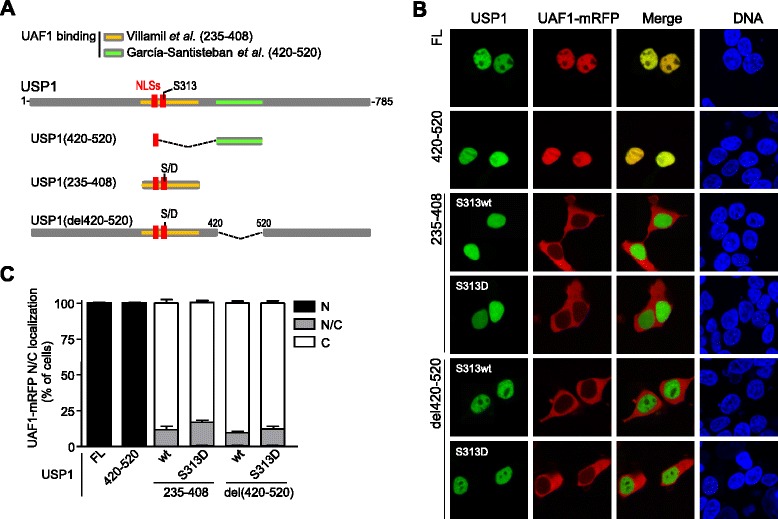
**Side-by-side comparison of two reported UAF1-binding sites in USP1 using a nuclear relocation assay. A**. Schematic representation of USP1 and the deletion mutants used in the analysis. The two reported UAF1 binding sites are highlighted: in orange, the 235–408 segment reported by Villamil *et al.* [17]; in green, the 420–520 segment reported by García-Santisteban *et al*. [18]. The location of USP1 nuclear localization signals (NLSs, in red), and the S313 phosphorylation site is also shown. S313 wild type and S313D phosphomimetic mutants of USP1(235–408) and USP1(del420-520) were used. **B**. Confocal images showing representative examples of the results using the *in vivo* nuclear relocation assay. 293T cells were co-transfected with UAF1-mRFP and GFP-USP1 full length (FL), GFP-USP1(420–520), YFP-USP1(235–408), YFP-USP1(235–408)^S313D^, YFP-USP1(del420-520) and YFP-USP1(del420-520)^S313D^. Cells were counterstained with Hoechst to show the nuclei (DNA panels). UAF1-mRFP clearly relocates to the nucleus when co-expressed with GFP-USP1 full length and GFP-USP1(420–520), but not with the remaining deletion mutants. **C**. Graph showing the results of a semiquantitative analysis of the nuclear relocation assay samples. Slides were coded and the nuclear (N), nuclear/cytoplasmic (N/C) or cytoplasmic (C) localization of UAF1-mRFP was determined in at least 100 cells per slide. The results (mean and SEM) of three independent experiments are shown in the graph.

The identity of the UAF1 binding region(s) in USP1 is a matter of some controversy (Figure 3A). Using the relocation assay and co-IP, the UAF1-binding site has been mapped to a motif comprising USP1 residues 420–520 [18]. On the other hand, using *in vitro* binding assays with purified recombinant proteins, UAF1 has been reported to bind USP1 amino acid segment 235–408, which corresponds to the non-conserved domain inserted between boxes 2 and 3 of USP1 [17]. The 235–408 segment includes USP1 nuclear localization signals (NLSs) and the S313 phosphorylation site. Importantly, only the phosphomimetic S313D mutant version of this segment was found to interact with UAF1 *in vitro* [17]. In an attempt to gauge the contribution of these two USP1 regions to UAF1-binding in a cellular context, we carried out a side-by-side test to compare USP1(420–520) and USP1(235–408) in the UAF1-relocation assay. S313 wild type and S313D phosphomimetic versions of USP1(235–408) were tested. In addition, S313 wild type and S313D versions of a fragment USP1(del420-520), lacking the 420–520 motif, were also included in the test.

YFP- or GFP-tagged versions of these USP1 variants were co-expressed with UAF1-mRFP in 293T cells. Full-length GFP-USP1 was used as a positive control. As described before [18], one of USP1 NLSs was used to ensure nuclear accumulation of the 420–520 motif. Figure 3B shows representative examples of the results obtained in the nuclear relocation assay. The percentage of cells showing nuclear (N), nuclear/cytoplasmic (N/C) or cytoplasmic (C) localization of UAF1-mRFP was determined in cells co-expressing the different GFP-USP1 variants, and the results of three independent assays are represented in Figure 3C. In line with our previous findings [18], full-length USP1 and the USP1(420–520) fragment induced a complete relocation of UAF1-mRFP to the nucleus. In contrast, UAF1-mRFP remained largely cytoplasmic when co-expressed with the other USP1 variants tested, including USP1(235–408)^S313D^ and USP1(del420-520)^S313D^. These results indicate that USP1 amino acid motif 420–520 is both necessary and sufficient for UAF1 binding in a cellular environment.

The failure of USP1(235–408)^S313D^ to induce UAF1-mRFP nuclear relocation contrasts with the reported ability of this fragment to interact with UAF1 *in vitro* [17]. These contrasting findings might result from competition between UAF1 and other proteins for binding to this USP1 fragment, an event that may occur in cellular assays but not in *in vitro* assays with purified proteins. Since the 235–408 motif overlaps with USP1 NLSs, we hypothesized that competition with the nuclear import machinery might prevent UAF1 binding to this motif in the cellular environment (Additional file 3A). To test this possibility, a variant of the USP1(235–408) fragment, termed USP1(235-408NLSm + NLS), was generated (Additional file 3A). This variant bears inactivating mutations in USP1 NLSs [18], and its nuclear localization is mediated by a heterologous NLS from the SV40 large T antigen (SV40NLS) fused to its amino terminus. USP1(235-408NLSm + NLS) and the phosphomimetic USP1(235-408NLSm + NLS)^S313D^ remained unable to induce UAF1-mRFP relocation to the nucleus (Additional file 3B). The possibility that the NLS-inactivating mutations might simultaneously interfere with UAF1 binding cannot be formally ruled out, but these results suggest that the inability of USP1(235–408) to promote UAF1 relocation cannot be ascribed to competition with the nuclear import machinery.

### Homologous amino acid motifs, mapping to the Fingers sub-domain, mediate binding of USP1 and USP46 to UAF1

**Figure 4:**
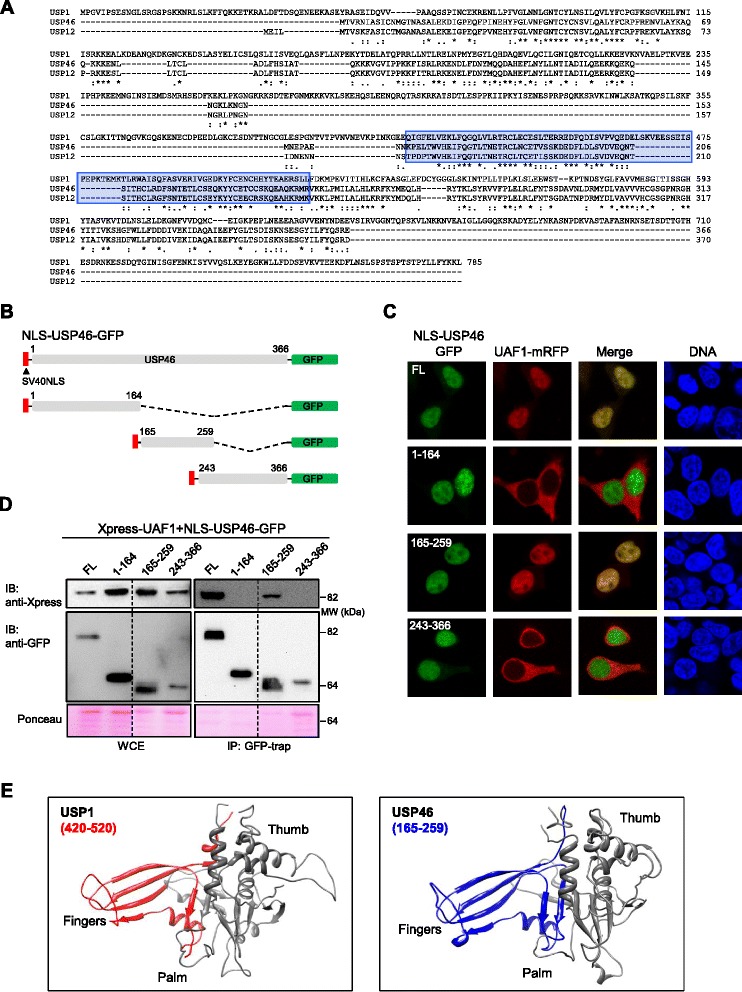
**Homologous amino acid motifs, mapping to the Fingers sub-domain, mediate binding of USP1 and USP46 to UAF1. A**. Alignment of USP1, USP46 and USP12 aminoacid sequences using CLUSTALW. USP1 aminoacid segment 420–520 and the homologous regions of USP46 and USP12 are highlighted in blue. **B**. Schematic representation of different GFP-tagged USP46 deletion mutants used to map its UAF1-binding motif. A heterologous nuclear localization signal (SV40 NLS) was fused to the amino terminal end of each fragment to ensure its nuclear accumulation **C**. Results of the UAF1 nuclear relocation assay with USP46 fragments. Confocal microscopy images show representative examples of 293T cells co-expressing UAF1-mRFP (red) and the different USP46 protein fragments (green). Cells were counterstained with Hoechst to show the nuclei (DNA panels). Nuclear relocation of UAF1-mRFP is induced by full length (FL) and the 165–259 fragment, but not by the 1–164 or the 243–366 fragments. **D**. Co-IP analysis of co-trasfected 293T cells, showing that Xpress-UAF1 co-immunoprecipitates with full-length USP46 and with the fragment encompassing residues 165–259, but not with the other two fragments tested. The dotted line indicates that the panel is a composite of two images from a single exposure of the same gel. WCE, whole cell extract. A section of the membrane stained with Ponceau is shown to gauge protein loading. **E**. Modeled structure of USP1 (left) and USP46 (right) catalytic domains using SWISS-MODEL and the USP7 structure 1NB8 [2] as a template. The Thumb, Palm and Fingers sub-domains are indicated. The UAF1-binding sites are highlighted in red (USP1 residues 420–520) or blue (USP46 residues 165–259).

In addition to USP1, two other DUBs, USP12 and USP46 interact with UAF1 [10], but the UAF1-binding sites in these DUBs have not yet been mapped. Considering the importance of UAF1 interaction for the enzymatic activity of these proteins [10,20], we reasoned that the critical UAF1-binding sequences would be conserved among these different DUBs. In order to test this possibility, and to gain further insight into UAF1/DUB interaction, we used CLUSTALW to align USP1, USP46 and USP12 amino acid sequences (Figure 4A) and BLAST to assess the similarity of USP1 fragments 235–408 and 420–520 with USP12 and USP46 (not shown). Not surprisingly, given that the 235–408 fragment maps to a non-conserved inserted domain [4], the degree of similarity of this fragment with USP12 (BLAST score 14.2) and USP46 (BLAST score 16.5) was markedly lower than that of the 420–520 fragment (BLAST score 52.0 with USP12 and 53.1 with USP46).

USP1 420–520 segment was found to be homologous to a central amino acid segment in USP12 and USP46. Therefore, we decided to carry out a deletion analysis followed by cell-based interaction assays to determine if this central motif mediates the binding of these other DUBs to UAF1. Since USP12 and USP46 are highly homologous proteins, we limited our analysis to USP46.

As shown in Figure 4B, three deletion mutants of USP46 tagged with GFP were generated: an amino-terminal fragment (1–164), a central fragment (165–259), encompassing the region of homology to USP1(420–520), and a carboxy-terminal fragment (243–266). In agreement with a previous report [21], we found that USP46-GFP is a cytoplasmic protein (Additional file 4). Thus, the SV40 NLS was fused to its amino terminus in order to force its nuclear import to carry out the UAF1-mRFP relocation assay (Additional file 4). This assay, (Figure 4C) as well as a subsequent co-IP analysis (Figure 4D), revealed that the UAF1-binding motif of USP46 lies within the 165–259 fragment. Thus, homologous amino acid sequences in USP1 and USP46 mediate binding to UAF1. Since the three-dimensional structure of USP1 and USP46 has not yet been reported, we used the SWISS-MODEL web tool [22] to model the structure of the catalytic domains of these DUBs using the structure of USP7 catalytic domain as a template. This model revealed that the UAF1 binding motifs of USP1 and USP46 map to their Fingers sub-domains (Figure 4E).

### Does autocleavage of USP1 at the G670/G671 motif occur in *cis* or in *trans*?

USP1 cleaves itself after the G670/G671 diglycine motif located near its carboxy-terminal end, within its second inserted domain. This autocleavage event produces two fragments that are subsequently degraded, and it was originally described as a mechanism that inactivates USP1 to allow for robust PCNA ubiquitination after UV-induced DNA damage [5]. Studies in chicken cells have shown that UV may also induce USP1 cleavage at other sites, which is not dependent on USP1 catalytic activity [23].

Previous reports have also shown that USP1 autocleavage at the G670/G671 motif can also occur in the absence of UV treatment [5,7]. In line with these findings, we noted (see Figures 1C and 2B) that GFP-USP1 wild type, but not the catalytically inactive mutant GFP-USP1^C90S^, readily undergoes cleavage when co-expressed with Xpress-UAF1 in 293T cells. This observation provides a convenient experimental system to further investigate USP1 autocleavage.

**Figure 5:**
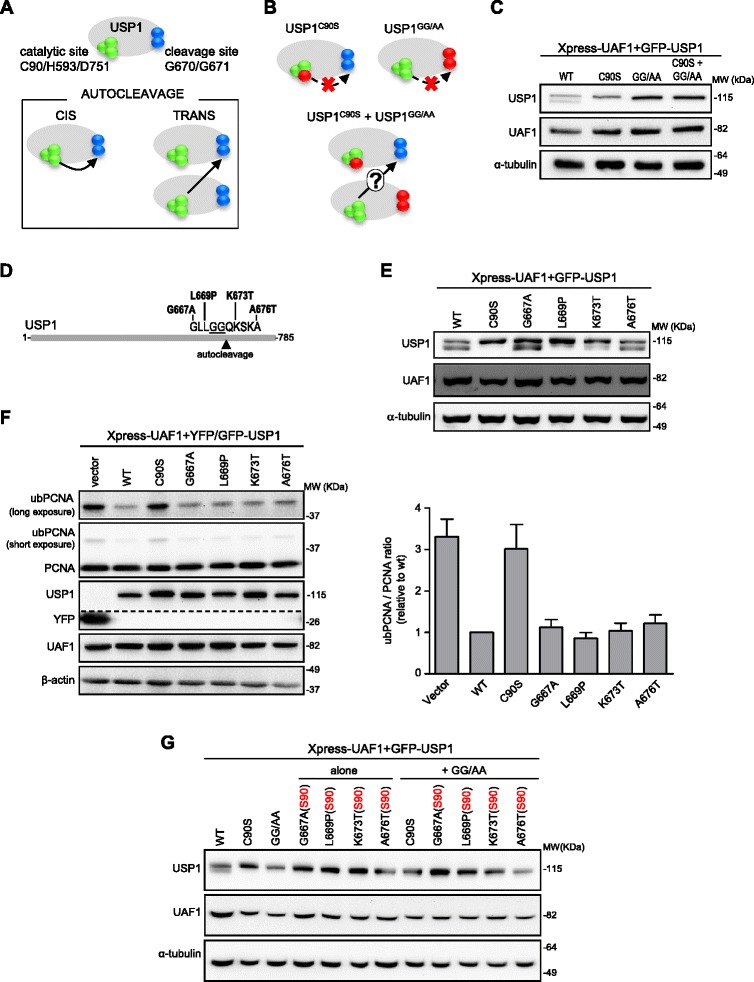
**USP1 autocleavage: assessing cis/trans mode of cleavage and the effect of cancer-associated mutations.** **A**. Representation of USP1 showing the catalytic triad (C90/H593/D751, green spheres) and the autocleavage site (G670/G671, blue spheres). Below, potential *cis*/*trans* autocleavage modes. **B**. Experiment used to assess cleavage mode. The C90S and GG/AA mutants (mutated residues in red) are unable to undergo *cis* autocleavage. If autocleavage occurs in *trans*, co-expression of both mutants could lead to cleavage of GFP-USP1^C90S^ by GFP-USP1^GG/AA^. **C**. Immunoblot analysis of cells expressing Xpress-UAF1 and GFP-USP1 constructs. Autocleavage was detected in cells transfected with wild type GFP-USP1, but not with GFP-USP1^C90S^ or GFP-USP1^GG/AA^. No cleavage was observed upon co-expression of GFP-USP1^C90S^ and GFP-USP1^GG/AA^, suggesting that *trans* cleavage does not occur. **D**. Location of cancer mutations [24] near USP1 autocleavage site. **E**. Immunoblot analysis of cells expressing Xpress-UAF1 and different USP1 mutants. GFP-USP1^G667A^, GFP-USP1^K673T^ and GFP-USP1^A676T^ are cleaved as efficiently as wild type USP1, whereas GFP-USP1^L669P^ is less efficiently cleaved. **F**. PCNA ubiquitination in 293T cells treated with 4 mM HU for 24 h. An example of immunoblot results is shown (left). The dotted line indicates that the panel is a composite of two images from a single exposure of the same gel. UbPCNA/PCNA ratio was determined by densitometry and the results (mean and SEM of 3 independent experiments) are shown in the graph (right). Cancer-associated mutations did not alter the ubPCNA/PCNA ratio. **G**. The C90S mutation (S90) was introduced into each cancer-related mutant construct, rendering these mutants unable to undergo *cis* autocleavage (Additional Figure 5C). These double mutants were expressed together with GFP-USP1^GG/AA^ to evaluate if they undergo *trans* cleavage. No autocleavage band was observed with any of the cancer-related/(S90) mutants upon co-expression with GFP-USP1^GG/AA^, suggesting that the cancer-related mutations tested do not alter the *cis*/*trans* mode of cleavage.

One of the aspects of USP1 autocleavage that remains to be elucidated is whether it occurs intramolecularly (in *cis*) or intermolecularly (in *trans*) (Figure 5A). The three-dimensional structure of USP1 has not yet been solved, and the autocleavage motif lies within a non-conserved domain whose structure cannot be reliably modelled using other USPs as template. Thus, it remains unknown if the cleavage site of a USP1 molecule can reach the catalytic site of the same molecule for the cleavage to occur in *cis*. In an attempt to shed some light on this issue, we devised the experiment illustrated in Figure 5B. A catalytically inactive mutant (USP1^C90S^), as well as a mutant with alanine substitutions at the G670/G671 cleavage site (USP1^GG/AA^) cannot undergo autocleavage in *cis*. However, it has been shown that USP1^GG/AA^ maintains its enzymatic activity [5,23] and, in fact, it efficiently deubiquitinates ubPCNA when transfected in 293T cells (Additional file 5A). Therefore, we reasoned that, if USP1 autocleavage occurs in *trans*, USP1^GG/AA^ molecules could cleave USP1^C90S^ molecules. To test this hypothesis, GFP-USP1^GG/AA^ and GFP-USP1^C90S^ were either individually or simultaneously co-expressed with Xpress-UAF1 in 293T cells. Wild type GFP-USP1 was used as a positive control. As shown in Figure 5C, simultaneous co-expression of GFP-USP1^GG/AA^ and GFP-USP1^C90S^ did not result in cleavage. It remains certainly possible that, in this experimental setting, GFP-USP1^GG/AA^ and GFP-USP1^C90S^ molecules did not come into close enough proximity for cleavage to occur in *trans* and, therefore, our results do not completely exclude the possibility of USP1 *trans* cleavage. However, our findings support the view that USP1 autocleavage occurs in *cis*.

### A cancer-associated missense mutation in USP1 hampers autocleavage

A second aspect about USP1 autocleavage that has not yet been investigated is the possibility that it could be altered by naturally occurring cancer-associated mutations. Although USP1 gene mutations are not a frequent event in human tumors, the Catalogue Of Somatic Mutations in Cancer (COSMIC) [24] database includes over thirty missense mutations in USP1, whose functional effect has not yet been tested. Four of these mutations (G667A, L669P, K673T and A676T) cluster within a ten amino acid segment encompassing the G670/G671 autocleavage site (Figure 5D). To determine if any of these cancer-associated mutations might alter USP1 cleavage, we used site-directed mutagenesis to introduce each of these amino acid changes into GFP-USP1, and we co-expressed these proteins with Xpress-UAF1 in 293T cells. It is important to note that none of these mutations disrupted USP1/UAF1 interaction, according to the results of a nuclear relocation assay (Additional file 5B). Immunoblot analysis (Figure 5E, and Additional file 5C) revealed that GFP-USP1^G667A^, GFP-USP1^K673T^ and GFP-USP1^A676T^ were cleaved to a similar extent as wild type GFP-USP1, while cleavage of the GFP-USP1^L669P^ mutant was much less efficient.

We next tested the ability of these cancer-associated USP1 mutants to deubiquitinate PCNA. As shown in Figure 5F, the four tested cancer-associated mutants readily decreased HU-induced PCNA ubiquitination in 293T cells. In contrast, cells transfected with the YFP vector or the catalytically inactive GFP1^C90S^ (negative controls) showed higher levels of ubPCNA.

Finally, we aimed to evaluate the possibility that the cancer-related mutants might alter the *cis/trans* mode of cleavage. To this end, we introduced the catalytic-site mutation C90S into each cancer-related mutant construct. As shown in Additional file 5C, the C90S mutation rendered these mutants unable to undergo autocleavage. These double mutants also failed to undergo cleavage when expressed in combination with GFP-USP1^GG/AA^ (Figure 5G), suggesting that the cancer-related mutations tested here do not alter the *cis/trans* mode of cleavage of USP1.

## Discussion

Since the discovery, nearly a decade ago, that USP1 plays an important role in the cellular response to DNA damage [5,25], significant advances have been made on the understanding of the function and regulation of this DUB. It has been shown, for example, that USP1 carries out its cellular activities in the context of a heterodimeric complex with UAF1, which enhances the stability and the enzymatic activity of the DUB [7,8]. The stability and activity of USP1 have been shown to be further regulated by several mechanisms, including CDK-mediated phosphorylation at the S313 residue [16] and cleavage by either itself [5] or other proteases [23,26].

The USP1/UAF1 complex is emerging as a novel target for cancer treatment (reviewed in [1]), and inhibitors of USP1 catalytic activity have been reported to reverse the resistance to platinum-based chemotherapeutic drugs in NSCLC cells [14,15] and to inhibit the growth of leukemic cell lines [27]. Further development of the therapeutic potential of this deubiquitinase complex would benefit from a better understanding of several aspects of its regulation that remain controversial, incompletely characterized or unexplored.

For example, protein-protein interactions are increasingly regarded as promising therapeutic targets in cancer [28] and thus, characterizing the molecular determinants of USP1/UAF1 interaction may be important to guide efforts aimed to disrupt this interaction with therapeutic purposes. In this regard, phosphorylation at the S313 residue has been recently reported to be critical for USP1 activity and interaction with UAF1 in an *in vitro* setting [17]. Here, we show that a non-phosphorylatable mutant USP1^S313A^ is still able to bind UAF1 when tested in cell-based assays. These assays were carried out using ectopically expressed proteins, but are, in our view, closer to a physiological setting than *in vitro* binding assays with purified proteins. In particular, the nuclear relocation assay with fluorescently-tagged proteins allows for visualization of the results in live cells. Of note, USP1/UAF1 interactions demonstrated using this assay could be consistently validated by co-IP in the present study, as well as in our previous report [18]. Importantly, our data indicate that USP1^S313A^ forms a functional complex with UAF1 in transfected cells. This complex is able to reverse monoubiquitination of endogenous PCNA, a well-established cellular substrate of USP1 [5,15,26,29]. Our results do not definitely exclude the possibility that S313 phosphorylation may modulate the activity of the USP1/UAF1 complex. In fact, minor, but reproducible differences in the levels of ubPCNA were noted between cells expressing wild type USP1, USP1^S313A^ or a phosphomimetic USP1^S313D^ mutant. In line with previous *in vitro* results using an artificial fluorogenic substrate [17], PCNA deubiquitination appeared to be more efficient in cells expressing the S313D phosphomimetic USP1. Nevertheless, S313 phosphorylation does not seem to be an essential requisite for USP1 activity or UAF1 binding in a cellular environment.

Our finding that phosphorylation of USP1 at S313 is dispensable for UAF1 binding in cells, led us to use the relocation assay to directly compare the relative contribution of two proposed UAF1-binding sites in USP1: the 235–408 fragment containing the S313 residue [17], and the 420–520 fragment [18]. In marked contrast to the 420–520 fragment, the 235–408 fragment (in either the S313 wild type or phosphomimetic forms) was unable to promote the nuclear relocation of UAF1-mRFP. Furthermore, deletion of the 420–520 fragment abrogated UAF1 relocation, regardless of the presence of the S313D phosphomimetic mutation. Thus, USP1 420–520 amino acid motif is both necessary and sufficient for UAF1 binding in cells. It is important to note that this motif was not tested in the previous *in vitro* UAF1-binding assays [17].

The present findings seem to contradict previous *in vitro* results showing an interaction of the USP1(235–408)^S313D^ motif with UAF1. It must be taken into account, however, that many interacting partners of both USP1 and UAF1 may be present within the crowded environment of intact cells [30]. If USP1(235–408)^S313D^ interacts weakly with UAF1, a negative result in the nuclear relocation assay may be due to competition by endogenous partner(s). In this regard, nuclear import receptors (importins) were obvious candidates to compete with UAF1 for binding to USP1(235–408), because USP1 NLSs lie within this motif, and the importin KPNA1 has been identified as a potential USP1 interactor [30]. Although USP1 also bears a potential nuclear export sequence (NES) that could mediate interaction with the export receptor CRM1, the physiological relevance of this sequence remains to be established [13], and it lies outside the 235–408 segment. Importantly, NLS mutations do not increase nuclear relocation of UAF1-mRFP by USP1(235–408)^S313D^, suggesting that competition with importins does not account for the lack of interaction between USP1(235–408)^S313D^ and UAF1 in a cellular setting.

Altogether, our findings indicate that the USP1 420–520 fragment is the critical site for robust UAF1-binding in a cellular environment. In line with this view, the UAF1-binding site of USP46, another UAF1-interacting DUB [10], was mapped to amino acids 165–259, which is a motif homologous to USP1(420–520). The three-dimensional structure of USP1 and USP46 have not yet been solved, but *in silico* modeling using the structure of USP7 catalytic domain [2] as a template indicates that USP1(420–520) and USP46(165–259) UAF1-binding motifs lie within their Fingers sub-domain. Structural analyses have shown that the Fingers sub-domain contributes to ubiquitin binding by other members of the USP family [2,31,32]. Since UAF1 stimulates the deubiquitinating activity of USP1 and USP46 [7,10], we speculate that UAF1 and ubiquitin can simultaneously bind to opposite surfaces of the Fingers sub-domain in these DUBs.

Besides S313 phosphorylation, a well-established mechanism regulating USP1 is autocleavage at the G670/G671 diglycine motif [5]. Although originally reported to be induced by UV light [5], we could readily detect this cleavage event in 293T cells co-expressing GFP-USP1 and Xpress-UAF1. This observation provided a convenient experimental system to evaluate two aspects of USP1 autocleavage that have not been tested. On one hand, we have used co-expression of catalytically inactive (USP1^C90S^) and non-cleavable (USP1^GG/AA^) mutants to provide some experimental evidence suggesting that USP1 may undergo autocleavage in *cis.* Due to the technical limitations of our assay, this evidence is not conclusive. A conceptually similar *in vitro* test, co-incubating purified recombinant forms of USP1^C90S^ and USP1^GG/AA^, could provide further evidence. On the other hand, we have evaluated how USP1 autocleavage could be altered by mutations identified in human tumors.

To the best of our knowledge, no functional test on naturally-occurring USP1 mutations has yet been reported and thus, the effect that cancer-associated mutations may have on the function or regulation of USP1 remains unexplored. Around forty cancer-associated USP1 mutations were included in the COSMIC database by September 2013 [13]. Some of these changes were non-sense or frameshift mutations that would most likely result in a non-functional allele, but most USP1 mutations lead to single amino acid substitutions whose effect is still unknown. Four of these missense mutations, G667A, L669P, K673T and A676T, are located adjacent or in close proximity to USP1 autocleavage site, and our data indicate that one of these changes, the L669P mutation, decreases USP1 cleavage efficiency. This region of USP1 corresponds to a non-conserved inserted domain between boxes 5 and 6, and therefore, no structural information can be obtained by *in silico* modeling using other USPs as template. We hypothesize that this mutation might introduce a conformational change that hampers access of the cleavage site to the catalytic site. The USP1^L669P^ mutant clearly retains its ability to deubiquitinate PCNA in HU-treated cells. It is tempting to speculate that, by disrupting the normal balance of USP1 cleavage, this L699P mutation may contribute to tumorigenesis, and further experiments should address this possibility.

## Conclusions

In contrast to previous *in vitro* findings, or results indicate that USP1 phosphorylation at S313 is not critical for PCNA deubiquitination, neither for binding to UAF1 in a cellular environment. In this environment, USP1 amino acid motif 420–520 is necessary and sufficient for UAF1 binding, and thus represents a critical UAF1-binding site in USP1. This motif, and a homologous amino acid segment that mediates USP46 binding to UAF1, map to the Fingers sub-domain of these DUBs. Finally, our results support the view that USP1 autocleavage may occur in *cis,* and show that the balance of USP1 autocleavage can be disrupted by a cancer-associated mutation.

## Methods

### Plasmids, cloning procedures and site-directed mutagenesis

Plasmids encoding GFP-USP1 and Xpress-UAF1 were generously provided by Dr. Rene Bernards (Netherlands Cancer Institute, Amsterdam, The Netherlands) and Dr. Jae U. Jung (University of Southern California, Los Angeles, USA), respectively. Flag-HA-USP46 was obtained from the laboratory of Dr. John W. Harper (Harvard Medical School, Boston, USA) through Addgene (Plasmid #22584). YFP-USP1 (del420–520) and NLS-USP1(420–520)-GFP plasmids have been described previously [18].

To generate the plasmid encoding UAF1-mRFP, UAF1 cDNA was amplified by PCR and cloned in frame to mRFP using XhoI/AgeI restriction sites. On the other hand, a DNA sequence encoding USP1 amino acid segment 235–408 was amplified by PCR using GFP-USP1 as a template, and cloned as either a KpnI/BamHI fragment into pEYFP-C1 (Clontech), or as a BamHI/AgeI fragment into a vector termed pNLS(SV40)-GFP. The vector pNLS(SV40)-GFP derives from a NES-GFP plasmid previously used in a nuclear import assay [18]. Finally, DNA sequences encoding full length USP46 and the three deletion mutants (1–164, 165–259 and 243–366) were amplified by PCR using Flag-HA-USP46 as a template, and cloned as BamHI/AgeI fragments into pNLS(SV40)-GFP. Full-length USP46 was also cloned as a BamHI/AgeI fragment into a mutant version of pNLS(SV40)-GFP vector carrying a non-functional NLS sequence to generate the USP46-GFP plasmid. All PCR amplifications were carried out using high fidelity Pfu UltraII fusion HS DNA polymerase (Stratagene).

USP1 point mutations were created using the QuickChange Lightning Site-Directed Mutagenesis Kit (Stratagene), according to manufacturer’s directions.

All the new constructs generated were subjected to DNA sequencing (STABVIDA), and the absence of any unwanted mutation was confirmed. The sequences of the oligonucleotides used in cloning and site-directed mutagenesis are available upon request.

### Cell culture, transfection and drug treatment

Human embryonic kidney 293T (HEK293T) cells were grown in Dulbecco’s modified Eagle’s medium (Invitrogen), supplemented with 10% fetal bovine serum (Invitrogen), 100 U/ml penicillin and 100 μg/ml streptomycin (Invitrogen). Twenty four hours before transfection cells were seeded in 12-well or 6-well tissue culture plates or 10 cm petri dishes. Transfections were carried out with X-tremeGENE 9 transfection reagent (Roche Diagnostics) following manufacturer’s protocol.

Hydroxyurea (Sigma-Aldrich) was added to the culture medium 48 hours after transfection to a final concentration of 4 mM for 24 hours.

### Microscopy analysis of nuclear relocation assay samples

The nuclear relocation assay was carried out in cells seeded onto sterile glass coverslips. Cells co-expressing UAF1-mRFP with the different GFP- or YFP-tagged proteins were fixed with 3.7% formaldehyde in phosphate-buffered saline (PBS) for 30 min, incubated with Hoechst 33285 (Sigma) to visualize the nuclei, washed with PBS, and mounted onto microscope slides using Vectashield (Vector laboratories). Single-slice images were acquired using an Olympus Fluoview FV500 confocal microscope. Sequential acquisition of each fluorochrome was performed in order to avoid overlapping of fluorescent emission spectra. For for live cell imaging, cells were grown in 35 mm ibiTreat μ-dish slides (Ibidi), transfected with the indicated plasmids and examined using a Zeiss ApoTome.2 microscope. Semiquantitative analysis of nuclear relocation assay samples was carried out by determining the nucleocytoplasmic localization of UAF1-mRFP in at least 100 co-transfected cells per slide using a Zeiss Axioskop fluorescence microscope. Slides were coded to ensure unbiased scoring, and examined by two independent observers.

### Immunoblot analysis and co-immunoprecipitation

Cells were washed with ice-cold PBS and collected in lysis buffer containing 10 mM sodium phosphate (pH 7.4), 150 mM NaCl, 1 mM EDTA, 1 mM EGTA, 10 mM β-glycerophosphate, 0.5% NP40, 10 mM phenylmethylsulfonyl fluoride, 10 mM sodium orthovanadate, 10 μg/ml protease inhibitor cocktail (Roche), and 50 mM N-ethylmaleimide (Thermo Scientific). Protein concentration was determined using the DC Protein Assay (Bio-Rad). For immunoblot analysis, protein samples were resolved in 8%, 10% or 12% SDS-PAGE and transferred onto nitrocellulose membranes (Bio-Rad). Prior to antibody incubation, membranes were stained with Ponceau to assess protein loading. Membranes were blocked with 5% non-fat dry milk in TTBS and incubated with the primary antibodies: anti-GFP (Chromotek, 1:1000), anti-Xpress (Invitrogen, 1:5000), anti-PCNA (Santa Cruz, 1:400), anti-β-actin (Sigma-Aldrich, 1:3000) and anti-α-tubulin (Sigma-Aldrich, 1:3000). Subsequently, membranes were incubated with the corresponding horseradish peroxidase-conjugated secondary antibodies (Santa cruz, 1:3000), and developed with ECL or Femto chemiluminiscence reagents (Thermo Scientific). Semiquantive analysis of immunoblot bands was performed by densitometry using Quantity One software 4.6 (Bio-Rad Laboratories).

Immunoprecipitation of GFP- or YFP-fusion proteins was carried out using Magnetic GFP-Trap beads (Chromotek), following manufacturer’s directions. Immunoprecipitated proteins were analysed by immunoblot as described above.

## Additional files

Additional file 1:
**A. Cytoplasmic localization of UAF1-mRFP expressed alone.** Image showing that UAF1-mRFP is cytoplasmic when expressed alone in 293T cells. This finding suggests that endogenous levels of USP1 are not sufficient to mediate relocation of ectopically expressed UAF1-mRFP to the nucleus. B, C. Effect of USP1 siRNA on GFP-USP1-mediated relocation of UAF1-mRFP. The graph shows the results of a quantitative RT-PCR analysis to measure endogenous USP1 mRNA level in 293T transfected with control or USP1-targeted siRNAs. Cells were transfected with scramble siRNA (C(−)) or a pool of three siRNAs targeting USP1 (Ambion, Life Technologies) using Lipofectamine RNAiMAX transfection reagent (Life Technologies). Total RNA was isolated using High Pure RNA Isolation Kit (Roche Diagnostics), and complementary DNA (cDNA) was synthesized using the High Capacity cDNA Reverse Transcription Kit (Applied Biosystems). Quantitative real-time PCR (qRT-PCR) was performed using SYBR Premix Ex Taq (TaKaRa). Gene expression primers and probes (Integrated DNA Technologies) were used to specifically amplify USP1 and human GAPDH as an endogenous control. As shown in the graph, the silencing efficiency was approximately 80% at the mRNA level. Confocal images show the results of a UAF1-relocation assay in 293T cells first transfected with the corresponding siRNAs (scrambled, left panels; USP1, right panels), and subsequently with GPF-USP1WT, GFP-USP1S313A and GFP-USP1S313D. Under these conditions all GFP-USP1 variants were able to efficiently promote relocation of UAF1-mRFP to the nucleus. Additional file 2:
**Whole cell lysate immunoblot allows detection of PCNA monoubiquitination in response to hydroxyurea treatment.** Image of a complete western blot membrane probed with the anti-PCNA antibody PC10 (Santa Cruz Biotechnology Ref: sc-56). A clear single band of the correct size (approximately 30 kDa) is detected in untreated (UT) 293T cells. Following 24 h treatment with 4 mM hydroxyurea (HU), a second band, with a higher molecular weight (approximately 38 kDa, the expected size of monoubiquitinated PCNA) is readily detected. Additional file 3:
**A. Schematic representation of USP1 protein illustrating the hypothesis that UAF1 and importins might compete for binding to USP1(235–408).** The UAF1-binding motif reported by Villamil et al., (orange) including the S313 phosphorylation site, overlaps with USP1 NLSs (red). In order to test this hypothesis, the variant USP1(235-408NLSm + NLS) (represented below) was generated. In this variant, USP1 NLSs are inactivated, and its nuclear localization is mediated by an SV40 NLS fused to its amino terminus. S313 wild type and S313D phosphomimetic mutants of USP1(235-408NLSm + NLS) were used. B. Mutation of USP1 NLSs does not result in UAF1 binding to the 235–408 segment. Confocal images showing representative examples of the results using the *in vivo* nuclear relocation assay. 293T cells were co-transfected with UAF1-mRFP and YFP-USP1(235–408), GFP-USP1(235-408NLSm + NLS), and GFP-USP1(235-408NLSm + NLS)S313D. Cells were counterstained with Hoechst to show the nuclei (DNA panels). Additional file 4:
**Cytoplasmic USP46 can be targeted to the nucleus by the fusion of a heterologous NLS.** Confocal images show that Flag-HA-tagged and GFP-tagged USP46 (green panels) are localized in the cytoplasm of transfected 293T cells. However, amino-terminal fusion of the SV40 NLS sequence (PKKKRKV) leads to nuclear accumulation of USP46. The nucleocytoplasmic localization of UAF1-mRFP (red panels) parallels that of the DUB. Cells were counterstained with Hoechst to show the nuclei (DNA panels). Additional file 5:
**(A) The USP1GG/AA autocleavage site mutant deubiquitinates PCNA.** Immunoblot analysis of 293T cells co-transfected with Xpress-UAF1 and GFP-USP1 wild type (WT), the catalytically inactive GFP-USPC90S or the autocleavage site mutant GFP-USP1GG/AA. Cells were treated with 4 mM hydroxyurea (HU) for 24 h. Using an anti-PCNA antibody, monoubiquitinated PCNA (ubPCNA) is detected as a band migrating slightly above the non-ubiquitinated form. The image in the ubPCNA panel was obtained using a longer exposure time. The dotted line indicates that the panel is a composite of two images from a single exposure of the same gel. Expression of wild type GFP-USP1 or GFP-USP1GG/AA decreased PCNA monoubiquitination, whereas expression of GFP-USP1C90S did not. Expression of β-actin was used as a loading control. (B) Cancer-associated USP1 mutants relocate UAF1 to the nucleus. Confocal images showing representative examples of nuclear relocation assay results with four cancer-associated USP1 mutants. 293T cells were co-transfected with UAF1-mRFP and YFP vector (negative control), GFP-USP1G667A, GFP-USP1L669P, GFP-USP1K673T, and GFP-USP1A676T. Cells were counterstained with Hoechst to show the nuclei (DNA panels). The four mutants were able to induce the nuclear relocation of UAF1-mRFP, suggesting that the ability to interact with UAF1 is not disrupted by these mutations. (C) Mutation of the catalytic C90 residue abrogates autocleavage of USP1 cancer-related mutants. Immunoblot analysis of 293T cells co-transfected with different USP1 constructs and Xpress-UAF1. By introducing a C90S point mutation (S90), the autocleavage of USP1 cancer-related mutants was completely abrogated (note disappearance of the lower band). Note that the cleavage of L669P is *per se* very inefficient, and totally abrogated by the C90S mutation.

## Abbreviations

CDK: Cyclin dependent kinase
co-IP: Co-immunoprecipitation
DUB: Deubiquitinase
EDTA: Ethylenediaminetetraacetic acid
EGTA: Ethylene glycol tetraacetic acid
FL: Full length
GFP: Green fluorescent protein
HU: Hydroxyurea
IP: Immunoprecipitate
mRFP: Monomeric Red fluorescent protein
NES: Nuclear export signal
NLS: Nuclear localization signal
NSCLC: Non-small cell lung cancer
PCNA: Proliferating cell nuclear antigen
SEM: Standard error of the mean
SV40: Simian virus 40
TLS: Translesion synthesis
TTBS: Tween tris buffered saline buffer
UAF1: USP1-associated factor 1
Ub: Ubiquitin
USP: Ubiquitin specific protease
YFP: Yellow fluorescent protein
WCE: Whole cell extract
