# Supplementary material for: Structure-function analysis of USP1: insights into the role of Ser313 phosphorylation site and the effect of cancer-associated mutations on autocleavage
Source: Mol Cancer. 2015 Feb 6;14(1):33. doi: 10.1186/s12943-015-0311-7 (PMC4326527; doi:10.1186/s12943-015-0311-7)
Supplement: Additional file 1: — A. Cytoplasmic localization of UAF1-mRFP expressed alone. Image showing that UAF1-mRFP is cytoplasmic when expressed alone in 293T cells. This finding suggests that endogenous levels of USP1 are not sufficient to mediate relocation of ectopically expressed UAF1-mRFP to the nucleus. B, C. Effect of USP1 siRNA on GFP-USP1-mediated relocation of UAF1-mRFP. The graph shows the results of a quantitative RT-PCR analysis to measure endogenous USP1 mRNA level in 293T transfected with control or USP1-targeted siRNAs. Cells were transfected with scramble siRNA (C(−)) or a pool of three siRNAs targeting USP1 (Ambion, Life Technologies) using Lipofectamine RNAiMAX transfection reagent (Life Technologies). Total RNA was isolated using High Pure RNA Isolation Kit (Roche Diagnostics), and complementary DNA (cDNA) was synthesized using the High Capacity cDNA Reverse Transcription Kit (Applied Biosystems). Quantitative real-time PCR (qRT-PCR) was performed using SYBR Premix Ex Taq (TaKaRa). Gene expression primers and probes (Integrated DNA Technologies) were used to specifically amplify USP1 and human GAPDH as an endogenous control. As shown in the graph, the silencing efficiency was approximately 80% at the mRNA level. Confocal images show the results of a UAF1-relocation assay in 293T cells first transfected with the corresponding siRNAs (scrambled, left panels; USP1, right panels), and subsequently with GPF-USP1WT, GFP-USP1S313A and GFP-USP1S313D. Under these conditions all GFP-USP1 variants were able to efficiently promote relocation of UAF1-mRFP to the nucleus. [file 12943_2015_311_MOESM1_ESM.pptx]

## Slide 1
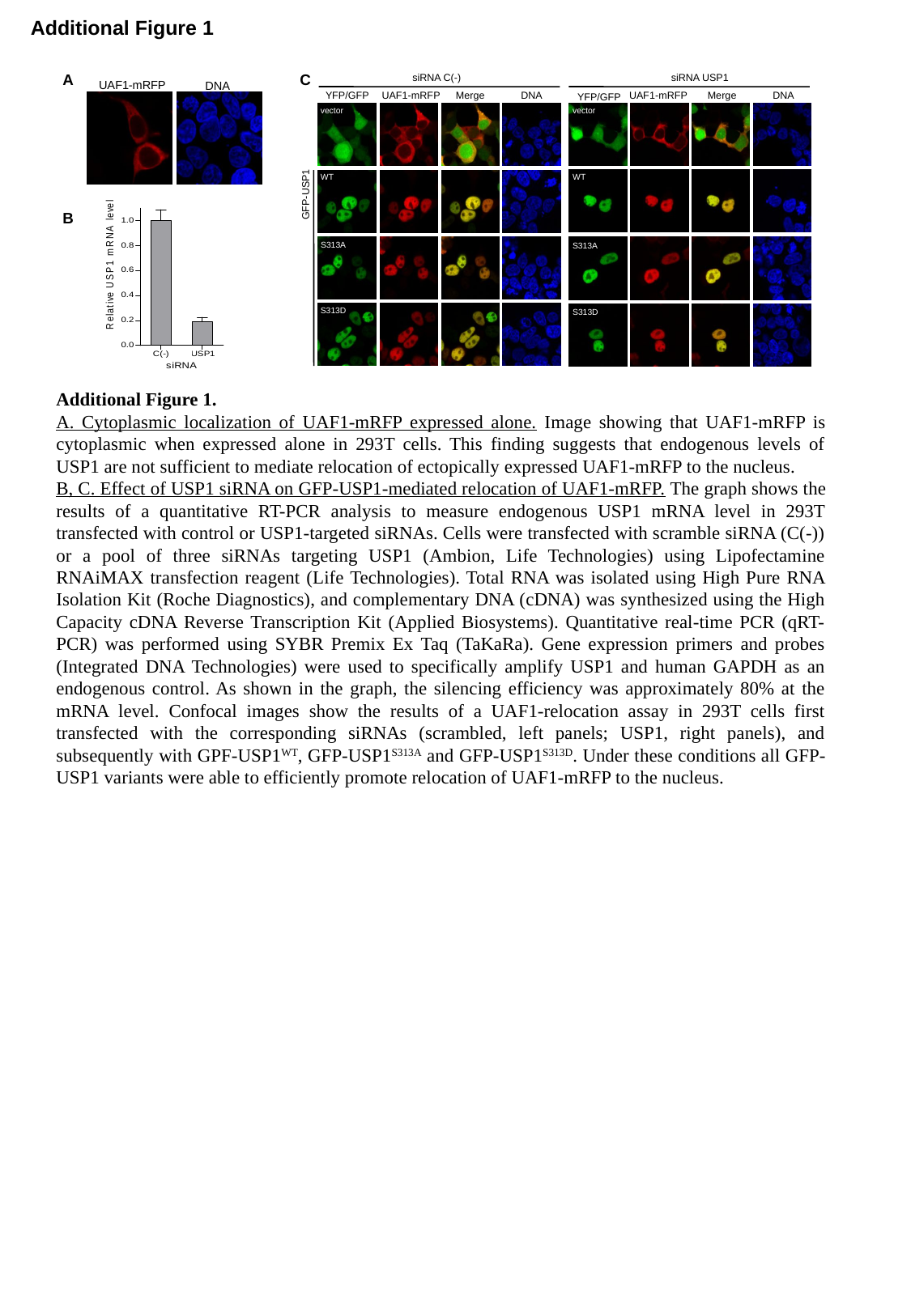

Additional Figure 1
A
C
siRNA USP1
siRNA C(-)
UAF1-mRFP
DNA
YFP/GFP
UAF1-mRFP
Merge
DNA
UAF1-mRFP
Merge
DNA
YFP/GFP
vector
vector
WT
WT
GFP-USP1
B
S313A
S313A
S313D
S313D
Additional Figure 1.
A. Cytoplasmic localization of UAF1-mRFP expressed alone. Image showing that UAF1-mRFP is cytoplasmic when expressed alone in 293T cells. This finding suggests that endogenous levels of USP1 are not sufficient to mediate relocation of ectopically expressed UAF1-mRFP to the nucleus.
B, C. Effect of USP1 siRNA on GFP-USP1-mediated relocation of UAF1-mRFP. The graph shows the results of a quantitative RT-PCR analysis to measure endogenous USP1 mRNA level in 293T transfected with control or USP1-targeted siRNAs. Cells were transfected with scramble siRNA (C(-)) or a pool of three siRNAs targeting USP1 (Ambion, Life Technologies) using Lipofectamine RNAiMAX transfection reagent (Life Technologies). Total RNA was isolated using High Pure RNA Isolation Kit (Roche Diagnostics), and complementary DNA (cDNA) was synthesized using the High Capacity cDNA Reverse Transcription Kit (Applied Biosystems). Quantitative real-time PCR (qRT-PCR) was performed using SYBR Premix Ex Taq (TaKaRa). Gene expression primers and probes (Integrated DNA Technologies) were used to specifically amplify USP1 and human GAPDH as an endogenous control. As shown in the graph, the silencing efficiency was approximately 80% at the mRNA level. Confocal images show the results of a UAF1-relocation assay in 293T cells first transfected with the corresponding siRNAs (scrambled, left panels; USP1, right panels), and subsequently with GPF-USP1WT, GFP-USP1S313A and GFP-USP1S313D. Under these conditions all GFP-USP1 variants were able to efficiently promote relocation of UAF1-mRFP to the nucleus.
