# Supplementary material for: Structure-function analysis of USP1: insights into the role of Ser313 phosphorylation site and the effect of cancer-associated mutations on autocleavage
Source: Mol Cancer. 2015 Feb 6;14(1):33. doi: 10.1186/s12943-015-0311-7 (PMC4326527; doi:10.1186/s12943-015-0311-7)
Supplement: Additional file 2: — Whole cell lysate immunoblot allows detection of PCNA monoubiquitination in response to hydroxyurea treatment. Image of a complete western blot membrane probed with the anti-PCNA antibody PC10 (Santa Cruz Biotechnology Ref: sc-56). A clear single band of the correct size (approximately 30 kDa) is detected in untreated (UT) 293T cells. Following 24 h treatment with 4 mM hydroxyurea (HU), a second band, with a higher molecular weight (approximately 38 kDa, the expected size of monoubiquitinated PCNA) is readily detected. [file 12943_2015_311_MOESM2_ESM.ppt]

## Slide 1
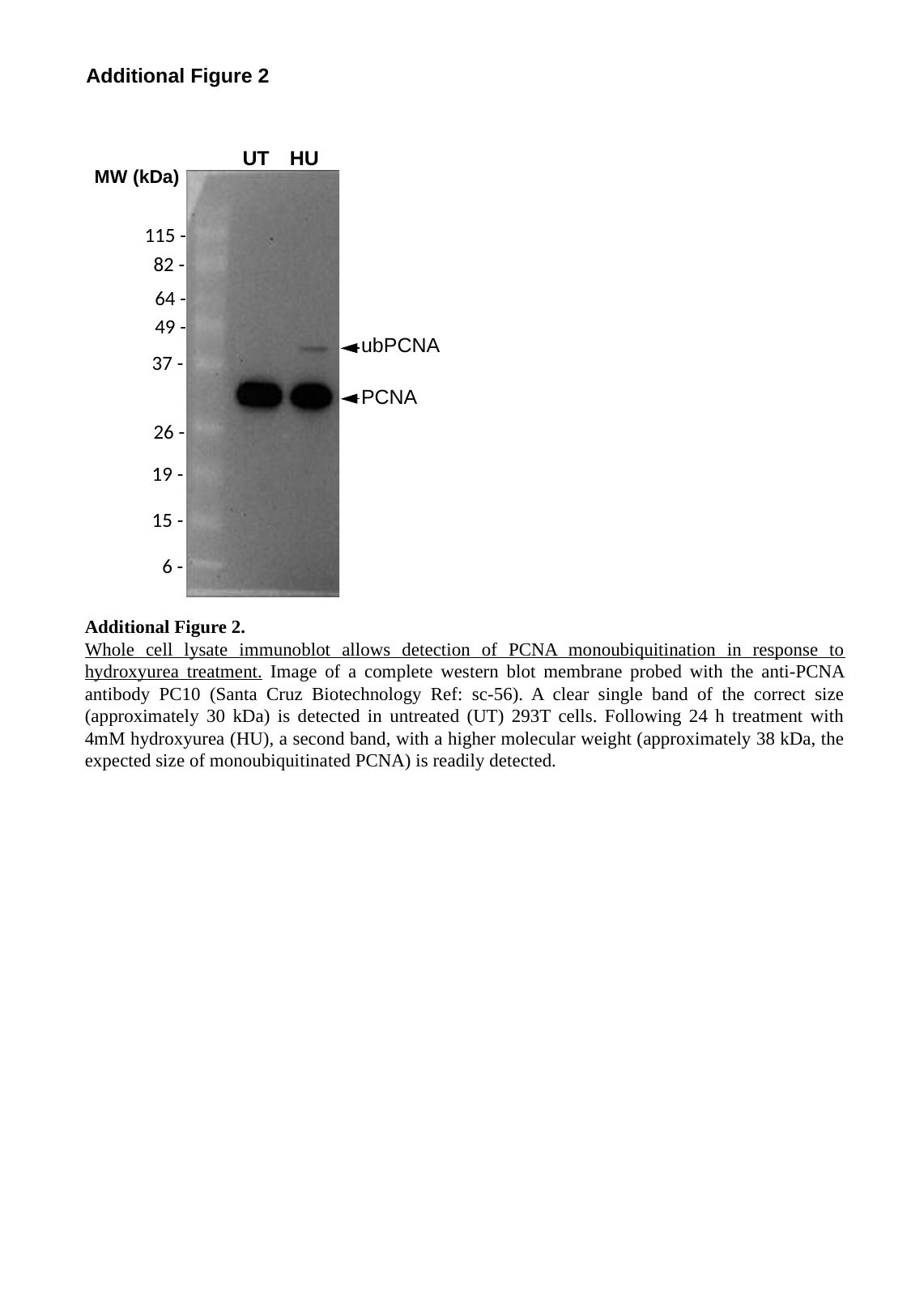

Additional Figure 2
UT
HU
MW (kDa)
115 -
82 -
64 -
49 -
ubPCNA
37 -
PCNA
26 -
19 -
15 -
6 -
Additional Figure 2.
Whole cell lysate immunoblot allows detection of PCNA monoubiquitination in response to hydroxyurea treatment. Image of a complete western blot membrane probed with the anti-PCNA antibody PC10 (Santa Cruz Biotechnology Ref: sc-56). A clear single band of the correct size (approximately 30 kDa) is detected in untreated (UT) 293T cells. Following 24 h treatment with 4mM hydroxyurea (HU), a second band, with a higher molecular weight (approximately 38 kDa, the expected size of monoubiquitinated PCNA) is readily detected.
