# Supplementary material for: Structure-function analysis of USP1: insights into the role of Ser313 phosphorylation site and the effect of cancer-associated mutations on autocleavage
Source: Mol Cancer. 2015 Feb 6;14(1):33. doi: 10.1186/s12943-015-0311-7 (PMC4326527; doi:10.1186/s12943-015-0311-7)
Supplement: Additional file 3: — A. Schematic representation of USP1 protein illustrating the hypothesis that UAF1 and importins might compete for binding to USP1(235–408). The UAF1-binding motif reported by Villamil et al., (orange) including the S313 phosphorylation site, overlaps with USP1 NLSs (red). In order to test this hypothesis, the variant USP1(235-408NLSm + NLS) (represented below) was generated. In this variant, USP1 NLSs are inactivated, and its nuclear localization is mediated by an SV40 NLS fused to its amino terminus. S313 wild type and S313D phosphomimetic mutants of USP1(235-408NLSm + NLS) were used. B. Mutation of USP1 NLSs does not result in UAF1 binding to the 235–408 segment. Confocal images showing representative examples of the results using the in vivo nuclear relocation assay. 293T cells were co-transfected with UAF1-mRFP and YFP-USP1(235–408), GFP-USP1(235-408NLSm + NLS), and GFP-USP1(235-408NLSm + NLS)S313D. Cells were counterstained with Hoechst to show the nuclei (DNA panels). [file 12943_2015_311_MOESM3_ESM.pptx]

## Slide 1
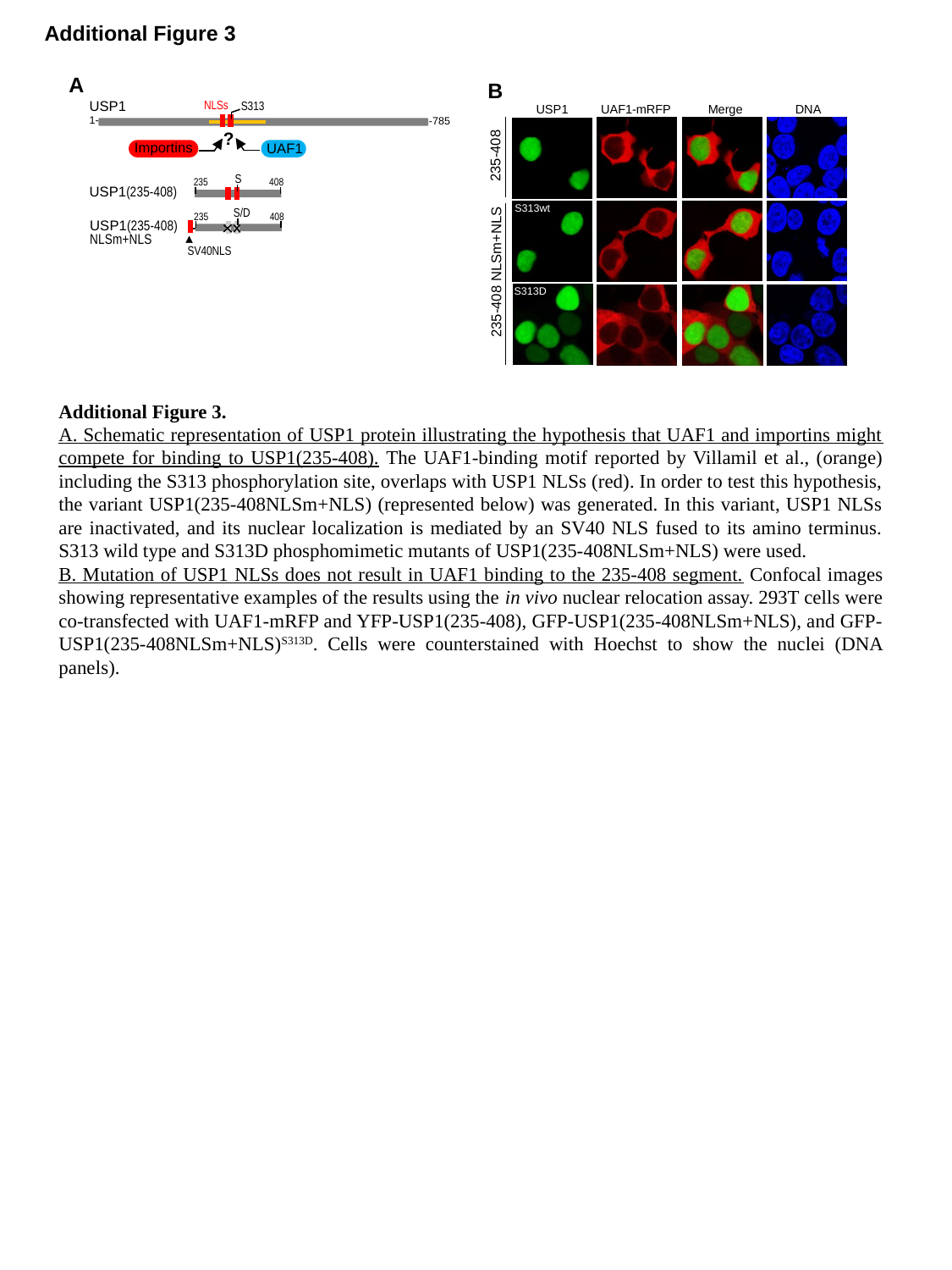

Additional Figure 3
A
B
USP1
NLSs
S313
USP1
UAF1-mRFP
Merge
DNA
1-
-785
?
Importins
UAF1
235-408
S
235
408
USP1(235-408)
S313wt
S/D
235
408
SV40NLS
USP1(235-408)
NLSm+NLS
235-408 NLSm+NLS
S313D
Additional Figure 3.
A. Schematic representation of USP1 protein illustrating the hypothesis that UAF1 and importins might compete for binding to USP1(235-408). The UAF1-binding motif reported by Villamil et al., (orange) including the S313 phosphorylation site, overlaps with USP1 NLSs (red). In order to test this hypothesis, the variant USP1(235-408NLSm+NLS) (represented below) was generated. In this variant, USP1 NLSs are inactivated, and its nuclear localization is mediated by an SV40 NLS fused to its amino terminus. S313 wild type and S313D phosphomimetic mutants of USP1(235-408NLSm+NLS) were used.
B. Mutation of USP1 NLSs does not result in UAF1 binding to the 235-408 segment. Confocal images showing representative examples of the results using the in vivo nuclear relocation assay. 293T cells were co-transfected with UAF1-mRFP and YFP-USP1(235-408), GFP-USP1(235-408NLSm+NLS), and GFP-USP1(235-408NLSm+NLS)S313D. Cells were counterstained with Hoechst to show the nuclei (DNA panels).
