# Supplementary material for: Structure-function analysis of USP1: insights into the role of Ser313 phosphorylation site and the effect of cancer-associated mutations on autocleavage
Source: Mol Cancer. 2015 Feb 6;14(1):33. doi: 10.1186/s12943-015-0311-7 (PMC4326527; doi:10.1186/s12943-015-0311-7)
Supplement: Additional file 4: — Cytoplasmic USP46 can be targeted to the nucleus by the fusion of a heterologous NLS. Confocal images show that Flag-HA-tagged and GFP-tagged USP46 (green panels) are localized in the cytoplasm of transfected 293T cells. However, amino-terminal fusion of the SV40 NLS sequence (PKKKRKV) leads to nuclear accumulation of USP46. The nucleocytoplasmic localization of UAF1-mRFP (red panels) parallels that of the DUB. Cells were counterstained with Hoechst to show the nuclei (DNA panels). [file 12943_2015_311_MOESM4_ESM.pptx]

## Slide 1
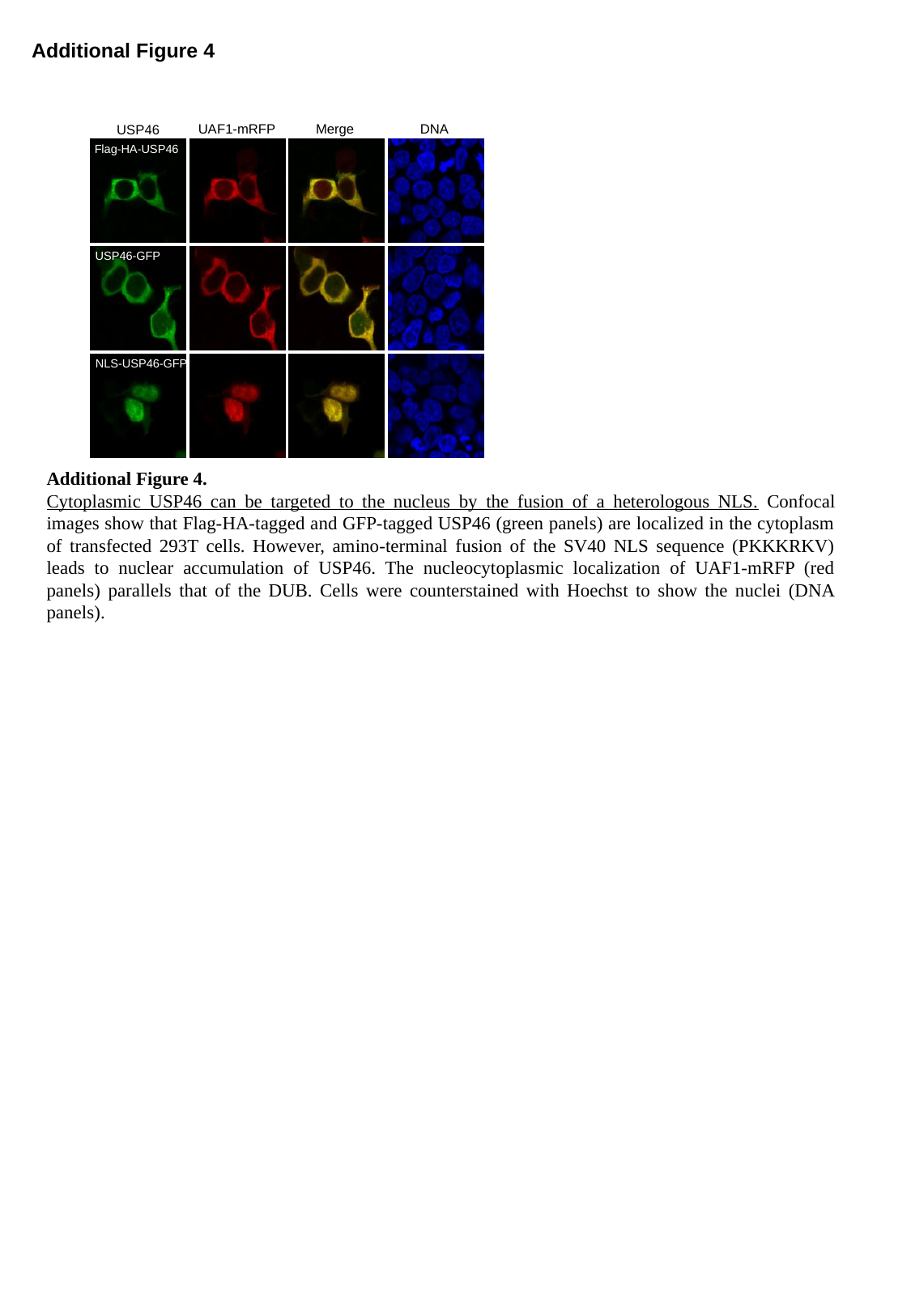

Additional Figure 4
UAF1-mRFP
Merge
DNA
USP46
Flag-HA-USP46
USP46-GFP
NLS-USP46-GFP
Additional Figure 4.
Cytoplasmic USP46 can be targeted to the nucleus by the fusion of a heterologous NLS. Confocal images show that Flag-HA-tagged and GFP-tagged USP46 (green panels) are localized in the cytoplasm of transfected 293T cells. However, amino-terminal fusion of the SV40 NLS sequence (PKKKRKV) leads to nuclear accumulation of USP46. The nucleocytoplasmic localization of UAF1-mRFP (red panels) parallels that of the DUB. Cells were counterstained with Hoechst to show the nuclei (DNA panels).
