# Supplementary material for: Structure-function analysis of USP1: insights into the role of Ser313 phosphorylation site and the effect of cancer-associated mutations on autocleavage
Source: Mol Cancer. 2015 Feb 6;14(1):33. doi: 10.1186/s12943-015-0311-7 (PMC4326527; doi:10.1186/s12943-015-0311-7)
Supplement: Additional file 5: — (A) The USP1GG/AA autocleavage site mutant deubiquitinates PCNA. Immunoblot analysis of 293T cells co-transfected with Xpress-UAF1 and GFP-USP1 wild type (WT), the catalytically inactive GFP-USPC90S or the autocleavage site mutant GFP-USP1GG/AA. Cells were treated with 4 mM hydroxyurea (HU) for 24 h. Using an anti-PCNA antibody, monoubiquitinated PCNA (ubPCNA) is detected as a band migrating slightly above the non-ubiquitinated form. The image in the ubPCNA panel was obtained using a longer exposure time. The dotted line indicates that the panel is a composite of two images from a single exposure of the same gel. Expression of wild type GFP-USP1 or GFP-USP1GG/AA decreased PCNA monoubiquitination, whereas expression of GFP-USP1C90S did not. Expression of β-actin was used as a loading control. (B) Cancer-associated USP1 mutants relocate UAF1 to the nucleus. Confocal images showing representative examples of nuclear relocation assay results with four cancer-associated USP1 mutants. 293T cells were co-transfected with UAF1-mRFP and YFP vector (negative control), GFP-USP1G667A, GFP-USP1L669P, GFP-USP1K673T, and GFP-USP1A676T. Cells were counterstained with Hoechst to show the nuclei (DNA panels). The four mutants were able to induce the nuclear relocation of UAF1-mRFP, suggesting that the ability to interact with UAF1 is not disrupted by these mutations. (C) Mutation of the catalytic C90 residue abrogates autocleavage of USP1 cancer-related mutants. Immunoblot analysis of 293T cells co-transfected with different USP1 constructs and Xpress-UAF1. By introducing a C90S point mutation (S90), the autocleavage of USP1 cancer-related mutants was completely abrogated (note disappearance of the lower band). Note that the cleavage of L669P is per se very inefficient, and totally abrogated by the C90S mutation. [file 12943_2015_311_MOESM5_ESM.pptx]

## Slide 1
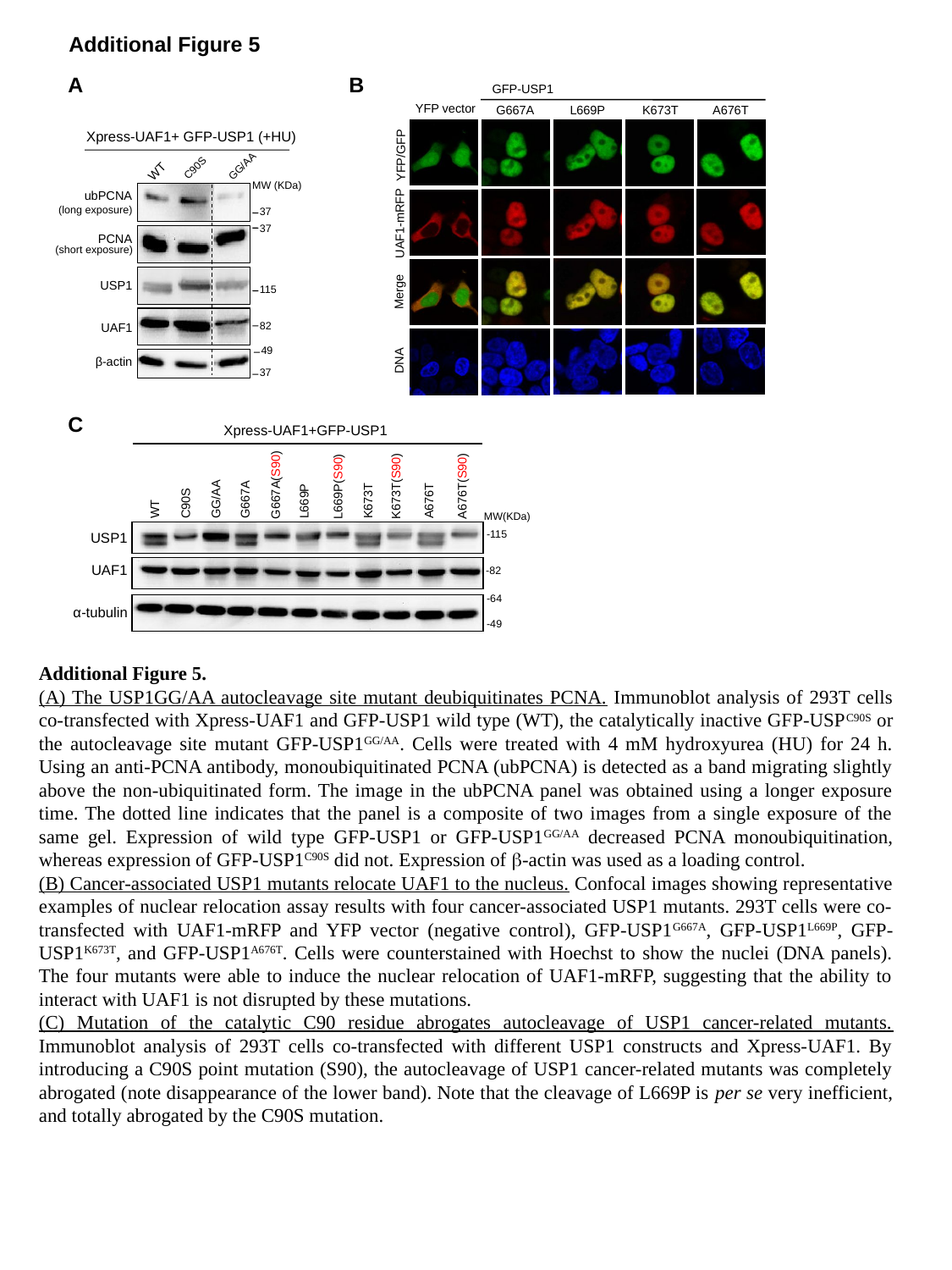

Additional Figure 5
A
B
GFP-USP1
YFP vector
G667A
L669P
K673T
A676T
Xpress-UAF1+ GFP-USP1 (+HU)
YFP/GFP
GG/AA
C90S
WT
MW (KDa)
ubPCNA
(long exposure)
37
UAF1-mRFP
37
PCNA
(short exposure)
USP1
115
Merge
82
UAF1
49
DNA
β-actin
37
C
Xpress-UAF1+GFP-USP1
G667A
G667A(S90)
A676T
A676T(S90)
L669P
L669P(S90)
K673T
K673T(S90)
GG/AA
C90S
WT
MW(KDa)
-115
USP1
UAF1
-82
-64
α-tubulin
-49
Additional Figure 5.
(A) The USP1GG/AA autocleavage site mutant deubiquitinates PCNA. Immunoblot analysis of 293T cells co-transfected with Xpress-UAF1 and GFP-USP1 wild type (WT), the catalytically inactive GFP-USPC90S or the autocleavage site mutant GFP-USP1GG/AA. Cells were treated with 4 mM hydroxyurea (HU) for 24 h. Using an anti-PCNA antibody, monoubiquitinated PCNA (ubPCNA) is detected as a band migrating slightly above the non-ubiquitinated form. The image in the ubPCNA panel was obtained using a longer exposure time. The dotted line indicates that the panel is a composite of two images from a single exposure of the same gel. Expression of wild type GFP-USP1 or GFP-USP1GG/AA decreased PCNA monoubiquitination, whereas expression of GFP-USP1C90S did not. Expression of b-actin was used as a loading control.
(B) Cancer-associated USP1 mutants relocate UAF1 to the nucleus. Confocal images showing representative examples of nuclear relocation assay results with four cancer-associated USP1 mutants. 293T cells were co-transfected with UAF1-mRFP and YFP vector (negative control), GFP-USP1G667A, GFP-USP1L669P, GFP-USP1K673T, and GFP-USP1A676T. Cells were counterstained with Hoechst to show the nuclei (DNA panels). The four mutants were able to induce the nuclear relocation of UAF1-mRFP, suggesting that the ability to interact with UAF1 is not disrupted by these mutations.
(C) Mutation of the catalytic C90 residue abrogates autocleavage of USP1 cancer-related mutants. Immunoblot analysis of 293T cells co-transfected with different USP1 constructs and Xpress-UAF1. By introducing a C90S point mutation (S90), the autocleavage of USP1 cancer-related mutants was completely abrogated (note disappearance of the lower band). Note that the cleavage of L669P is per se very inefficient, and totally abrogated by the C90S mutation.
